# Prosocial behavior predicts meaning in life during the COVID-19 pandemic: The longitudinal mediating role of perceived social support

**DOI:** 10.3389/fpubh.2023.1115780

**Published:** 2023-03-16

**Authors:** Yumei He, Qun Liu, Ofir Turel, Qinghua He, Shuyue Zhang

**Affiliations:** ^1^Department of Psychology, Faculty of Education, Guangxi Normal University, Guilin, China; ^2^Guangxi University and College Key Laboratory of Cognitive Neuroscience and Applied Psychology, Guangxi Normal University, Guilin, China; ^3^The School of Marxism, Neijiang Normal University, Neijiang, China; ^4^School of Computing and Information Systems, The University of Melbourne, Parkville, VIC, Australia; ^5^MOE Key Laboratory of Cognition and Personality, Faculty of Psychology, Southwest University, Chongqing, China; ^6^Ethnic Education Development Research Center of Guangxi Zhuang Autonomous Region, Guilin, China

**Keywords:** COVID-19, multidimensional prosocial behaviors, perceived social support, meaning in life, longitudinal mediating

## Abstract

The COVID-19 pandemic was an unexpected, long-term negative event. Meaning in life has been linked to better psychological adjustment to such events. The current study uses longitudinal data collected during the COVID-19 pandemic to discover whether perceived social support mediates the relationship between six dimensions of prosocial behavior (Altruistic, Anonymous, Public, Compliant, Emotional, and Dire) and meaning in life. A sample of Chinese college students (*N* = 514) was tracked at three time points (T1, T2, and T3) during the COVID-19 outbreak. A cross-lagged panel model (CLPM) was used for mediation analysis. The mediation effect was found in all the dimensions of prosocial behavior except for Public prosocial behavior. We also found a longitudinal, bidirectional association between perceived social support and meaning in life. The current study contributes to the growing literature on the significance of prosocial behavior in predicting meaning in life.

## 1. Introduction

### 1.1. Meaning in life

Humans are characterized by “a will to meaning” [(1), p.121]. Meaning in life has been defined as “the extent to which people comprehend, make sense of, or see significance in their lives” (2), which plays a promotive role in physical and mental health, longevity, resilience, life satisfaction, and in enhancing feelings of wellbeing (3–7). Meaninglessness, on the other hand, is a key component and/or driver of psychological distress, such as depression and suicide ideation, and even mental disorders, like neurosis and substance use disorders (8–11).

In many cases, the motivation to deal with the meaningfulness of one's own life comes from an upsetting or traumatic event. The prolonged COVID-19 pandemic is an example of such an event. It is a critical crisis that has affected every aspect of human life on a global scale. It can also cause panic reactions and mental health problems (12). Previous studies have suggested that having meaning in life may provide a psychological buffer that can help deal with adversity, such as COVID-19, and can therefore promote wellbeing and mental health (13–15). It has been a dire period for humanity as a whole, making it all the more important for individuals to reclaim control of their lives and regain meaning in life through action.

### 1.2. Meaning in life and six dimensions of prosocial behavior

Research has demonstrated a strong correlation between prosocial behavior and meaning in life. Studies have found that individuals with higher meaning in life are more likely to engage in prosocial activities, such as donating, writing gratitude notes, and self-reported altruistic behaviors (5, 16, 17). Moreover, the pursuit of meaning is associated with increased volunteering and donation behavior (16, 18). Although the relationship between prosocial behavior and meaning in life is evident, it is likely to be more complex, varying across individuals, cultures, and types of behavior. It is somewhat less clear how these effects occur and how multidimensional prosocial behaviors promote meaning in life. Prosocial behavior can comprise a vast array of behaviors, which can be classified in several ways. For instance, they can be classified by the motivations for prosocial behavior, which range from pure benevolence to reputational considerations and religious obligation (19, 20), or by the context: emergency situations vs. not, spontaneous vs. asked, and with or without onlookers (21). In the present study, we employed the Prosocial Tendencies Measure (PTM), which suggests six dimensions of prosocial behavior (Anonymous, Public, Compliant, Altruistic, Dire, and Emotional) and facilitates measuring prosocial behavior in a variety of settings (22).

Anonymous prosocial behavior is characterized as assistance rendered without the awareness of the recipient, which is unlikely to be driven by others' approval or rewards. The performer cannot expect concrete or social rewards (21), reflecting minimized self-interest (23). When players were aware that they could exert anonymous prosocial influence through gameplay, they enjoyed greater meaning (24).

Public prosocial behavior is the inclination to undertake prosocial behaviors in front of others, frequently driven by the desire for a particular reward. It is likely to be primarily egoistically motivated (25), which can be a manipulative interpersonal strategy for certain benefits or external regulation. For example, Public prosocial behavior is linked with parental use of social and material rewards (26). Children who have a strong desire to gain adult approval are more likely to engage in Public prosocial behavior (21). Public prosocial behavior is negatively related to sympathy (27) and positively related to narcissism (28, 29) and other dark traits (30). Unlike other prosocial behavior, Public prosocial behavior is likely to be negatively associated with meaning in life.

Compliant prosocial behavior is described as helping others in response to a verbal or non-verbal request, with a focus on spontaneously emitted vs. requested prosocial behavior. Compliant, like Anonymous, is primarily other-oriented, concentrating on the needs and conditions of beneficiaries (20, 29). Nonetheless, several scholars have questtioned the motivations for compliant prosocial behavior. Eisenberg suggested, for instance, that compliant prosocial behavior reflects a non-assertive personal style, internalized ideals, or a prosocial self-image rather than other-orientation and empathy/sympathy prosocial motives (31, 32). Some researchers have argued that compliant prosocial behavior demonstrates the actor's willingness to socially conform and engage in normative social behaviors in response to interpersonal norms (33). Therefore, it is less clear how Compliant affects meaning in life.

Altruistic prosocial behavior is defined as voluntary help motivated primarily by concern for the needs and welfare of another or by internalized morals (22). Altruists attempt to benefit others without seeking reward, but this attitude eventually rewards the performer with wellbeing and self-transcendence (34–36). Self-transcending and other-oriented behavior have been defined as the innate desire to discover meaning in human life. It broadens self-centered perspectives, connecting the self to the outside world (17, 37), which contributes to existential wellbeing (a strong purpose in life) (38).

Dire and Emotional prosocial behavior are two dimensions that are closely related to helping others in emergency or emotionally-charged circumstances, which are activated by cues from the social context. This connection has been observed across different cultural backgrounds (22, 39). Helping in emotionally evocative and/or emergency situations was assumedly related to sympathy and other-oriented personal tendencies (22, 40). However, few studies have discussed how Dire and Emotional prosocial behaviors affect meaning in life. Emotional, Compliant, and Dire are largely context-based and might have a subtle and less clear relationship with meaning in life.

### 1.3. Prosocial behavior and perceived social support

Perceived social support is a concept that refers to an individual's perception of the availability and satisfaction of support from significant others (41). This interpersonal coping resource develops over time as a two-way process between the individual and those around them (42). Studies have found that a responsive and supportive system can provide individuals with a sense of worth and protection and foster confidence and motivation (43). It has been linked to various positive outcomes, such as physical health, buffering of depression, life satisfaction, and wellbeing. On the other hand, poor-quality support can have a detrimental effect and exacerbate stress (16).

Research has demonstrated that prosocial behavior can lead to positive feedback from adults and peers, thus increasing interpersonal connections and strengthening relationships (22, 44). Teenagers are more likely to help friends than strangers, as helping is largely driven by a desire to maintain bonds and socioemotional connections (45). Studies have shown that perceived social support is strongly correlated with prosocial behavior (46). Halbesleben and Wheeler (47) proposed that helping others fosters social support by creating reciprocal relationships. Prosocial behavior can enhance a recipient's perceived social support, motivating them to reciprocate in order to maintain a balance between the amount of social support they receive and give (48). Thus, helping others is likely to be rewarded with social support from others (49). As such, we hypothesize that prosocial behavior is positively associated with perceived social support.

### 1.4. Perceived social support and meaning in life

No matter the culture, relationships are of great importance to all individuals. Certain cultures may prioritize social connections more than others (50). For instance, those in collectivistic societies, such as China, may prioritize social interaction more (51). The ability to form and maintain meaningful relationships is a major factor in determining quality of life (52). For instance, quality of relationships and interpersonal intimacy with significant others (i.e., family and friends) and a sense of belonging, connectedness, closeness, and social support (3, 53–58) all contribute to enhancing meaning in life. Social relationships are essential for organizing our experiences, providing our lives with purpose, and allowing our lives to have significance (59). The thriving through relationships model explains that social support is an interpersonal process that encourages thriving (60). When relational support is present, individuals are more likely to thrive despite hardships. Research has found that adults who report greater support and less strain in their social relationships are more likely to increase their sense of purpose over time (61). Conversely, social exclusion and ostracism can lead to a feeling of life being meaningless (3, 55). Moreover, the relationship between perceived social support and meaning in life is likely to be reciprocal. A nationally representative longitudinal study by Stavrova and Luhmann (58) found that the relationship between social connectedness and life meaning is bidirectional. Furthermore, a longitudinal study involving elderly people revealed that meaning in late life is associated with the social support received from family and close friends (3).

### 1.5. Perceived social support as a mediator

The pursuit of understanding one's experiences in relation to a greater context is a common endeavor associated with gaining a sense of purpose in life (Reker and Chamberlain, 2000). Research has indicated that engaging in prosocial behavior can be an effective coping mechanism during global adversity, as it can benefit both the individual and the wider society (46). Through helping others, individuals can gain a sense of belonging in the broader social order. Additionally, participating in a reciprocal social network can increase the perception of social support, which provides emotional and motivational regulation to assist in the life review process and cultivate a sense of personal value and influence (2, 17).

The essential virtue of Chinese culture, with Confucian culture at its center, is “benevolence” or “humanity” (ren), which means loving others (62). It contains overtones of self-love, and this self-love extends to all members of society. Ren embodies thoughts and values applicable to the entirety of human society (63). According to an old proverb, good is repaid with good, and wicked with evil. This is a fundamental and universal principle in Chinese culture. To love others and achieve a balance between the inner and outer worlds will ultimately lead to a rewarding existence (64).

Previous research has yet to explore the role of perceived social support as a mediator between prosocial activity and life meaning. While some studies have found partial mediation effects of other forms of social connection, such as relationship satisfaction with a close friend, they have not observed a significant mediating effect of social connectedness. However, the limitations of experiments and cross-sectional data, such as their propensity to produce biased estimates of longitudinal parameters (65), may have prevented a more comprehensive understanding of the relationship between prosocial behavior and meaning in life. Therefore, a longitudinal study is necessary to further investigate this link and its potential cultural nuances.

### 1.6. The present study

This study aims to explore how different variables changed before, during, and after the COVID-19 pandemic and the association between six dimensions of prosocial behavior, perceived social support, and meaning in life. Furthermore, the study seeks to investigate whether perceived social support mediates the relationship between the six dimensions of prosocial behavior and meaning in life.

We hypothesize that, during the COVID-19 pandemic, prosocial behavior increased, and perceived social support and meaning in life saw a decline. Additionally, we hypothesize that, except for Public, the other five dimensions of prosocial behavior are positively associated with meaning in life. Furthermore, we hypothesize that the relationship between perceived social support and meaning in life is bidirectional, and that perceived social support mediates the relationship between meaning in life and Anonymous, Compliant, Altruistic, Dire, and Emotional prosocial behavior, but not Public prosocial behavior.

## 2. Materials and methods

### 2.1. Participants

We conducted a web-based survey on college students recruited from a university in Sichuan, China. Data were gathered in three waves: November 2019, February 2020, and July 2020. Participation was voluntary and students were provided course credits for their involvement. Students were asked to answer questions regarding their situation in the past week. The initial sample size was 581, but 563 completed all three surveys (about 3% loss). Additionally 49 participants were excluded for failing attention check questions or giving monolithic responses (who poorly completed surveys or gave the same answer to all the questions), resulting in a final sample of 514 (403 women, Mage = 21 ± 1.01). Little's (66) missing completely at random (MCAR) test revealed significant missing data, χ^2^ (2198) = 2445.499, *p* < 0.001, which was handled using full information maximum likelihood estimates (FIML) procedure (67). All procedures and instruments used obtained approval from the local university's Institutional Review Board.

### 2.2. Measures

#### 2.2.1. Prosocial behavior

The Chinese version of the Prosocial Tendencies Measure (PTM) was used to measure six dimensions of prosocial behaviors (Altruistic, Compliant, Emotional, Dire, Public, and Anonymous) (22, 68). The PTM asked participants to rate 26 items on a five-point Likert scale. The Cronbach's αs in the present study were 0.912 for T1, 0.946 for T2, and 0.957 for T3.

#### 2.2.2. Perceived social support

Perceived social support was measured using the Perceived Social Support Scale (PSSS) (69, 70). Which was used to measure perceived support from family, friends, and significant others. PSSS is a 24-item scale employing a 5-point Likert-type format. Perceived social support was tested at T2 and T3, and Cronbach's α coefficients were 0.951 for T2 and 0.957 for T3.

#### 2.2.3. Meaning in life

Meaning in life was measured using the Chinese version of the Meaning in Life Questionnaire (MLQ-C) (71), which is a seven-point self-rating scale containing 10 items and divided into two dimensions: the presence of and the search for meaning in life (72). Previous work has shown that because of the independence of these two constructs, they can be assessed separately (8). Note that we focus on the presence of meaning. Therefore, we used the presence of meaning in life (MIL-p) dimension, which contains five questions. The Cronbach's α coefficients were 0.754 for T1; 0.775 for T2 and 0.760 for T3.

### 2.3. Data analysis

SPSS 21 was used to estimate descriptive statistics, correlations, and repeated measures analysis of variance (RM-ANOVA). RM-ANOVA were employed to examine mean differences across time. Secondly, confirmatory factor analysis (CFA) was used to evaluate the longitudinal measurement invariance for all measures across three measurement occasions. Changes in the ΔCFI that did not exceed a threshold of 0.01 and changes in ΔRMSEA that did not exceed a threshold of 0.015 were considered indicative of invariant measurement (73).

**Figure 1:**
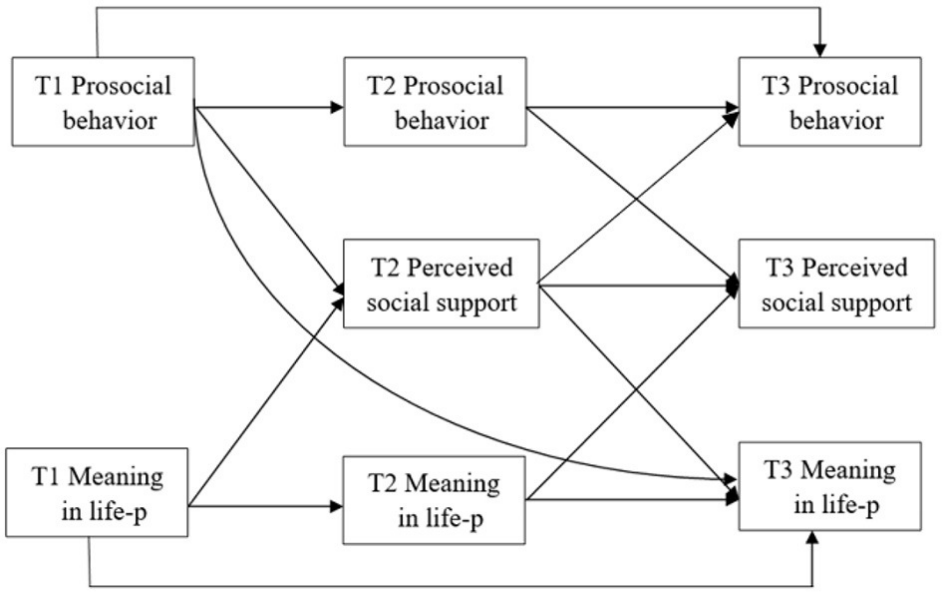
Our model tested how each dimension of prosocial behavior affects perceived social support and meaning in life. The arrows indicate the paths whereby each construct at Time t predicts the other constructs at Time t+1, with correlations omitted for simplicity.

Separate mediational analyses were conducted for each of the six dimensions of prosocial behavior using a structural equation model in Amos 24. We leveraged the longitudinal mediation from a cross-lagged panel model (CLPM) (74). The CLPM allows time for causes to have their effects, supports stronger inference about the direction of causation in comparison to models using cross-sectional data, and reduces the probable parameter bias that arises when using cross-sectional data (75). Cole and Maxwell (74) suggested that researchers should test for the presence of omitted paths in the model. In order not to miss any important paths, we used mode 1 (see Figure 1) to test the longitudinal relationship between different dimensions of prosocial behaviors, perceived social support, and the presence of meaning in life. All cross-lag paths occurred over one unit of time while considering the stability of all variables except for perceived social support (i.e., we controlled for prior levels of meaning in life when testing the association between perceived social support at T2 and meaning in life at T3). Due to the lack of data on perceived social support at T1, we did not control for prior levels of perceived social support at T1 when testing the association between prosocial behavior at T1 and perceived social support at T2. All variables or residuals at a given time point were allowed to correlate (i.e., the correlations between the different variables at each time point). Finally, the bias-corrected bootstrap test (using 5000 bootstrap resamples) was conducted to further evaluate the significance and CIs of the mediating effects (76, 77). This non-parametric analysis method is robust to violations of data normality conditions and produces a 95% confidence interval around the indirect effect. When this interval excludes zero, the indirect effect is considered significant (76). Standardized estimates are reported.

The following goodness-of-fit indices were used: Chi-square ratio (χ^2^/df), the chi-square test of significance, Comparative Fit Index (CFI), and Tucker-Lewis Index (TLI), with 0.95 or higher represents a good fit; Root Mean Square Error of Approximation (RMSEA) of 0.08 or lower represents a good fit, and the standardized root mean square residual (SRMR) and SRMR values <0.08 indicate good fit to the data (78).

## 3. Results

### 3.1. Descriptive statistics and correlations

Table 1 presents an overview of the means, standard deviations, and correlations of all study variables across the three waves. Anonymous, Compliant, Altruistic, Emotional, and Dire were statistically significant and positively correlated with PSS and MIL-p across time. Perceived social support was positively correlated with MIL-p across time. T1 Public prosocial behavior, however, was not significantly correlated with T2 Anonymous (*p* = 0.104), T2 Altruistic (*p* = 0.258), T3 Anonymous (*p* = 0.979), T3 Altruistic (*p* = 0.643), T3 Dire (*p* = 0.943), T3 PSS (*p* = 0.208), T2 MIL-p (*p* = 0.054) and T3MIL-p (*p* = 0.152); T2 Public prosocial behavior was not significantly correlated with T3 PSS (*p* = 0.206) and T1 MIL-p (*p* = 0.362); T3 Public prosocial behavior was not significantly correlated with T3 PSS (*p* = 0.293) and T1 MIL-p (*p* = 0.093). In addition, the correlation between Public prosocial behavior and the rest of the variables at other time points was significant.

**Table 1:** Means, standard deviations, and Bivariate correlations for all study variables.

	**1**	**2**	**3**	**4**	**5**	**6**	**7**	**8**	**9**	**10**	**11**	**12**	**13**	**14**	**15**	**16**	**17**	**18**	**19**	**20**	**21**	**22**	**23**
**1 T1 Anonymous**	1																						
**2 T1 Public**	0.263^***^	1																					
**3 T1 Compliant**	0.504^***^	0.419^***^	1																				
**4 T1 Altruistic**	0.677^***^	0.186^***^	0.574^***^	1																			
**5 T1 Dire**	0.551^***^	0.272^***^	0.481^***^	0.642^***^	1																		
**6 T1 Emotional**	0.514^***^	0.484^***^	0.597^***^	0.538^***^	0.545^***^	1																	
**7 T2 Anonymous**	0.441^***^	0.072	0.274^***^	0.317^***^	0.273^***^	0.253^***^	1																
**8 T2 Public**	0.177^***^	0.444^***^	0.258^***^	0.106^*^	0.115^**^	0.256^***^	0.339^***^	1															
**9 T2 Compliant**	0.321^***^	0.171^***^	0.501^***^	0.321^***^	0.233^***^	0.325^***^	0.631^***^	0.463^***^	1														
**10 T2 Altruistic**	0.325^***^	0.050	0.287^***^	0.414^***^	0.285^***^	0.246^***^	0.713^***^	0.200^***^	0.649^***^	1													
**11 T2Dire**	0.253^***^	0.106^*^	0.259^***^	0.304^***^	0.336^***^	0.292^***^	0.628^***^	0.304^***^	0.636^***^	0.736^***^	1												
**12 T2 Emotional**	0.275^***^	0.268^***^	0.346^***^	0.260^***^	0.260^***^	0.433^***^	0.663^***^	0.559^***^	0.679^***^	0.612^***^	0.632^***^	1											
**13 T3 Anonymous**	0.396^***^	0.001	0.263^***^	0.315^***^	0.256^***^	0.237^***^	0.596^***^	0.152^***^	0.435^***^	0.503^***^	0.445^***^	0.396^***^	1										
**14 T3 Public**	0.227^***^	0.386^***^	0.227^***^	0.171^***^	0.176^***^	0.296^***^	0.242^***^	0.588^***^	0.273^***^	0.159^***^	0.197^***^	0.389^***^	0.371^***^	1									
**15 T3 Compliant**	0.304^***^	0.137^**^	0.445^***^	0.283^***^	0.183^***^	0.305^***^	0.459^***^	0.257^***^	0.607^***^	0.473^***^	0.416^***^	0.478^***^	0.706^***^	0.458^***^	1								
**16 T3 Altruistic**	0.324^***^	0.020	0.333^***^	0.383^***^	0.265^***^	0.253^***^	0.497^***^	0.113^*^	0.470^***^	0.604^***^	0.463^***^	0.413^***^	0.771^***^	0.274^***^	0.732^***^	1							
**17 T3 Dire**	0.240^***^	−0.003	0.252^***^	0.288^***^	0.279^***^	0.276^***^	0.492^***^	0.152^***^	0.452^***^	0.544^***^	0.530^***^	0.477^***^	0.719^***^	0.326^***^	0.669^***^	0.774^***^	1						
**18 T3 Emotional**	0.272^***^	0.197^***^	0.316^***^	0.242^***^	0.239^***^	0.410^***^	0.450^***^	0.355^***^	0.461^***^	0.415^***^	0.418^***^	0.563^***^	0.695^***^	0.558^***^	0.744^***^	0.675^***^	0.721^***^	1					
**19 T2 PSS**	0.176^***^	0.092^*^	0.215^***^	0.213^***^	0.185^***^	0.228^***^	0.348^***^	0.133^**^	0.364^***^	0.414^***^	0.371^***^	0.315^***^	0.241^***^	0.047	0.292^***^	0.335^***^	0.316^***^	0.249^***^	1				
**20 T3 PSS**	0.185^***^	0.056	0.198^***^	0.244^***^	0.201^***^	0.236^***^	0.377^***^	0.056	0.345^***^	0.406^***^	0.352^***^	0.290^***^	0.466^***^	0.158^***^	0.438^***^	0.529^***^	0.509^***^	0.418^***^	0.511^***^	1			
**21 T1MIL-p**	0.314^***^	0.150^***^	0.289^***^	0.351^***^	0.290^***^	0.277^***^	0.192^***^	0.040	0.201^***^	0.247^***^	0.165^***^	0.172^***^	0.164^***^	0.074	0.182^***^	0.250^***^	0.203^***^	0.159^***^	0.269^***^	0.290^***^	1		
**22 T2MIL-p**	0.187^***^	0.085	0.162^***^	0.228^***^	0.130^**^	0.210^***^	0.306^***^	0.121^**^	0.302^***^	0.362^***^	0.278^***^	0.244^***^	0.238^***^	0.090^*^	0.225^***^	0.264^***^	0.226^***^	0.192^***^	0.442^***^	0.370^***^	0.496^***^	1	
**23 T3MIL-p**	0.208^***^	0.063	0.200^***^	0.266^***^	0.199^***^	0.204^***^	0.331^***^	0.121^**^	0.321^***^	0.345^***^	0.273^***^	0.288^***^	0.370^***^	0.157^***^	0.321^***^	0.408^***^	0.386^***^	0.355^***^	0.354^***^	0.550^***^	0.475^***^	0.539^***^	1
**M**	18.38	12.85	17.94	15.46	11.65	18.05	18.52	12.56	18.09	16.00	11.91	18.29	18.41	12.54	17.94	15.67	11.79	18.19	60.06	59.57	25.52	25.24	25.04
**SD**	2.66	2.71	2.73	2.24	1.65	2.72	3.65	3.30	3.41	2.80	2.04	3.48	3.71	3.24	3.57	3.00	2.21	3.69	12.02	12.47	4.53	4.61	4.67

*** p < 0.001,

** p < 0.005,

* p < 0.05,

PSS: perceived social support, MIL-p: the presence of meaning in life.

### 3.2. Studied variables change with the rise and fall of the COVID-19 pandemic

Altruistic at T2 was higher than T1 and T3, *F*(2, 1026) = 10.162, *p* < 0.001, η^2^ = 0.019. Dire at T2 was higher than T1 and T3, *F*_(2,1026)_ = 3.631, *p* < 0.05, η^2 =^ 0.007. Compliant [*F*_(2,1026)_ = 0.739, *p* = 0.476, η^2^ = 0.001], Emotional [*F*_(2,1026)_ = 1.178, *P* = 0.308, η^2^ = 0.002] and Anonymous [*F*_(2,1026)_ = 0.504, *P* = 0.604, η^2^ = 0.001] also showed an increase at T2, but the increase didn't approach significant. Public constantly decreased from T1 to T3, *F*_(2,1026)_ = 3.042, *p* < 0.05, η^2^ = 0.006. Perceived social support decreased from T2 to T3, but the difference was not statistically significant [*F*_(1,513)_ = 0.829, *p* = 0.363, η^2^ = 0.002]. MIL-p declined from T1 to T3, *F*_(2,1026)_ = 2.803, *p* = 0.061, η^2^ = 0.005. This result supports our first hypothesis, namely that During COVID-19, prosocial behavior increased, and perceived social support and meaning in life declined.

### 3.3. Longitudinal measurement invariance

Confirmatory factor analysis (CFA) demonstrated strong invariance (see Table 2), suggesting that observed changes in these constructs over time were meaningful rather than reflecting measurement artifacts or item biases (79).

**Table 2:** Comparison for longitudinal measurement invariance.

**Variables**	**Model tested**	**CFI**	**TLI**	**RMSEA**	**ΔCFI**	**ΔRMSEA**
Prosocial behavior	Configural invariance	0.952	0.934	0.076		
	Metric invariance	0.952	0.939	0.073	0	0.003
	**Scalar invariance**	**0.948**	**0.940**	**0.072**	**0.004**	**0.001**
Perceived social support	Configural invariance	0.993	0.988	0.076		
	Metric invariance	0.993	0.990	0.068	0	0.008
	**Scalar invariance**	**0.993**	**0.990**	**0.068**	**0**	**0**
Meaning in life	Configural invariance	0.973	0.960	0.054		
	Metric invariance	0.971	0.962	0.053	0.002	0.001
	**Scalar invariance**	**0.959**	**0.952**	**0.060**	**0.012**	**−0.007**

The bold values indicate established model of measurement invariance.

### 3.4. Longitudinal mediation effect

Table 3 shows the model fit of all six cross-lagged panel models and presents excellent fit indicators. Table 4 shows the standardized stability and cross-lagged coefficients. Table 5 shows the total effect, direct effect, and indirect effect of the six models.

**Table 3:** Model fit of all six cross-lagged panel models.

**Predictor**	**χ^2^**	***df***	***p***	**CFI**	**TLI**	**RMSEA**	**SRMR**
Anonymous	28.107	7	0.000	0.984	0.936	0.077	0.041
Public	11.833	7	0.106	0.996	0.983	0.037	0.022
Compliant	21.742	7	0.003	0.989	0.956	0.064	0.037
Altruistic	26.039	7	0.000	0.986	0.944	0.073	0.051
Dire	18.056	7	0.012	0.991	0.964	0.055	0.039
Emotional	28.469	7	0.000	0.983	0.931	0.077	0.047

**Table 4:** Overview of the standardized stability and cross-lagged coefficients.

**Predictor**	**Autoregressive path**	**β**	***p***	**Cross-lagged path**	**β**	***p***
**Anonymous**						
	T1 Anonymous → T2Anonymous	0.433	^***^	T1 Anonymous → T2 PSS	0.092	^*^
	T2 Anonymous → T3Anonymous	0.485	^***^	T1 Anonymous → T3 MIL-_P_	0.042	0.239
	T1 Anonymous → T3Anonymous	0.172	^***^	T2 Anonymous → T3 PSS	0.151	^***^
	T2 PSS → T3 PSS	0.403	^***^	T2 PSS → T3 Anonymous	0.045	0.227
	T1 MIL-_P_ → T2 MIL-_P_	0.486	^***^	T2 PSS → T3 MIL-_P_	0.132	^***^
	T2 MIL-_P_ → T3 MIL-_P_	0.358	^***^	T1 MIL-_P_ → T2 PSS	0.223	^***^
	T1 MIL-_P_ → T3 MIL-_P_	0.223	^***^	T2 MIL-_P_ → T3 PSS	0.134	^***^
**Public**						
	T1 Public → T2 Public	0.443	^***^	T1 Public → T2 PSS	0.048	0.231
	T2 Public → T3 Public	0.518	^***^	T1 Public → T3 MIL-_P_	−0.023	0.486
	T1 Public → T3 Public	0.156	^***^	T2 Public → T3 PSS	−0.05	0.132
	T2 PSS → T3 PSS	0.440	^***^	T2 PSS → T3 Public	−0.037	0.304
	T1 MIL-_P_ → T2 MIL-_P_	0.499	^***^	T2 PSS → T3 MIL-_P_	0.135	^***^
	T2 MIL-_P_ → T3 MIL-_P_	0.370	^***^	T1 MIL-_P_ → T2 PSS	0.265	^***^
	T1 MIL-_P_ → T3 MIL-_P_	0.227	^***^	T2 MIL-_P_ → T3 PSS	0.175	^***^
**Compliant**						
	T1 Compliant → T2 Compliant	0.496	^***^	T1Compliant → T2 PSS	0.147	^***^
	T2 Compliant → T3 Compliant	0.475	^***^	T1Compliant → T3 MIL-_P_	0.04	0.257
	T1 Compliant → T3 Compliant	0.185	^***^	T2Compliant → T3 PSS	0.110	^**^
	T2 PSS → T3 PSS	0.405	^***^	T2 PSS → T3Compliant	0.081	^*^
	T1 MIL-_P_ → T2 MIL-_P_	0.485	^***^	T2 PSS → T3 MIL-_P_	0.126	^**^
	T2 MIL-_P_ → T3 MIL-_P_	0.369	^***^	T1 MIL-_P_ → T2 PSS	0.209	^***^
	T1 MIL-_P_ → T3 MIL-_P_	0.219	^***^	T2 MIL-_P_ → T3 PSS	0.153	^***^
**Altruistic**						
	T1 Altruistic → T2 Altruistic	0.398	^***^	T1Altruistic → T2 PSS	0.123	^**^
	T2 Altruistic → T3 Altruistic	0.489	^***^	T1Altruistic → T3 MIL-_P_	0.061	0.089
	T1 Altruistic → T3 Altruistic	0.140	^***^	T2Altruistic → T3 PSS	0.164	^***^
	T2PSS → T3PSS	0.384	^***^	T2 PSS → T3Altruistic	0.106	^**^
	T1 MIL-_P_ → T2 MIL-_P_	0.474	^***^	T2 PSS → T3 MIL-_P_	0.125	^**^
	T2 MIL-_P_ → T3 MIL-_P_	0.366	^***^	T1 MIL-_P_ → T2 PSS	0.188	^***^
	T1 MIL-_P_ → T3 MIL-_P_	0.210	^***^	T2 MIL-_P_ → T3 PSS	0.138	^***^
**Dire**						
	T1 Dire → T2 Dire	0.339	^***^	T1Dire → T2 PSS	0.128	^**^
	T2 Dire → T3 Dire	0.434	^***^	T1Dire → T3 MIL-_P_	0.044	0.210
	T1 Dire → T3 Dire	0.086	^**^	T2Dire → T3 PSS	134	^***^
	T2 PSS → T3 PSS	0.400	^***^	T2 PSS → T3Dire	0.142	^***^
	T1 MIL-_P_ → T2 MIL-_P_	0.485	^***^	T2 PSS → T3 MIL-_P_	0.130	^***^
	T2 MIL-_P_ → T3 MIL-_P_	0.367	^***^	T1 MIL-_P_ → T2 PSS	0.211	^***^
	T1 MIL-_P_ → T3 MIL-_P_	0.211	^***^	T2 MIL-_P_ → T3 PSS	0.145	^***^
**Emotional**						
	T1 Emotional → T2 Emotional	0.423	^***^	T1 Emotional → T2 PSS	0.140	^***^
	T2 Emotional → T3 Emotional	0.439	^***^	T1 Emotional → T3 MIL-_P_	0.006	0.861
	T1 Emotional → T3 Emotional	0.187	^***^	T2 Emotional → T3 PSS	0.070	0.054
	T2 PSS → T3 PSS	0.417	^***^	T2 PSS → T3 Emotional	0.072	0.058
	T1 MIL-_P_ → T2 MIL-_P_	0.490	^***^	T2 PSS → T3 MIL-_P_	0.132	^***^
	T2 MIL-_P_ → T3 MIL-_P_	0.366	^***^	T1 MIL-_P_ → T2 PSS	0.218	^***^
	T1 MIL-_P_ → T3 MIL-_P_	0.229	^***^	T2 MIL-_P_ → T3 PSS	0.164	^***^

* p < 0.05;

** p < 0.005;

*** p < 0.001.

**Table 5:** Total, direct, and indirect effects of six dimensions of prosocial behavior to meaning in life.

	**β**	***SE***	***p***	**95%CI**
**Total model**				
T1 Anonymous→ T3 MIL_−P_	0.054	0.036	0.137	[−0.017, 0.125]
T1 Public→ T3 MIL_−P_	−0.017	0.035	0.621	[−0.084, 0.052]
T1 Compliant→ T3 MIL_−P_	0.058	0.033	0.071	[−0.006, 0.124]
T1 Altruistic→ T3 MIL_−P_	0.076	0.037	0.041	[0.003, 0.148]
T1 Dire→ T3 MIL_−P_	0.060	0.035	0.088	[−0.008, 0.131]
T1 Emotional→ T3 MIL_−P_	0.024	0.036	0.471	[−0.046, 0.098]
**Direct model**				
T1 Anonymous→ T3 MIL_−P_	0.042	0.036	0.230	[−0.028, 0.114]
T1 Public→ T3 MIL_−P_	−0.023	0.034	0.500	[−0.090, 0.044]
T1 Compliant→ T3 MIL_−P_	0.040	0.033	0.218	[−0.025, 0.107]
T1 Altruistic→ T3 MIL_−P_	0.061	0.037	0.134	[−0.012, 0.134]
T1 Dire→ T3 MIL_−P_	0.044	0.035	0.234	[−0.025, 0.112]
T1 Emotional→ T3 MIL_−P_	0.006	0.037	0.218	[−0.066, 0.080]
**Indirect model**				
T1 Anonymous→ T2 PSS→ T3 MIL_−P_	0.012	0.007	0.016	[0.002, 0.030]
T1 Public→ T2 PSS→ T3 MIL_−P_	0.006	0.006	0.167	[−0.003, 0.022]
T1 Compliant→ T2 PSS→ T3 MIL_−P_	0.018	0.008	0.003	[0.006, 0.041]
T1 Altruistic→ T2 PSS→ T3 MIL_−P_	0.015	0.008	0.005	[0.004, 0.037]
T1 Dire→ T2 PSS→ T3 MIL_−P_	0.017	0.008	0.003	[0.005, 0.037]
T1 Emotional→ T2 PSS→ T3 MIL_−P_	0.018	0.008	0.003	[0.006, 0.040]

For Anonymous, the direct effect was not significant, β = 0.042, *p* = 0.230, 95%CI = [−0.028, 0.114]. The indirect effect was statistically significant, β = 0.012, *p* < 0.05, 95%CI = [0.002, 0.030]; T1 Anonymous to T3 MIL-p was not significant (β = 0.042, *p* = 0.239), T2 PSS to T3 Anonymous was not significant (β = 0.045, *p* = 0.227).

For Public, the direct effect was not significant, β = −0.023, *p* = 0.500, 95%CI = [−0.090, 0.044]. The indirect effect was not significant, β = 0.006, *p* = 0.167, 95%*CI* = [−0.003, 0.022]. T1 Public to T2 PSS (β = 0.048, *p* = 0.231), T3 MIL-p (β = −0.023, *p* = 0.486) was not significant. T2 Public to T3 PSS was not significant (β = −0.05, *p* = 0.132), T2 PSS to T3 Public was not significant (β = −0.037, *p* = 0.304).

For Compliant, the direct effect was not significant, β = 0.040, *p* = 0.218, 95%CI = [−0.025, 0.107]. The indirect effect was statistically significant, β = 0.018, *p* < 0.005, 95% CI =[0.006, 0.041]. T1Compliant to T3 MIL-p was not significant (β = 0.04, *p* = 0.257).

For Altruistic, the direct effect was not significant, β = 0.061, *p*= 0.134, 95%CI = [−0.012, 0.134]. The indirect effect was statistically significant, β =0.015, *p* < 0.005, 95%CI = [0.004, 0.037]. T1Altruistic to T3 MIL-p was not significant (β = 0.061, *p* = 0.089).

For Dire, the direct effect was not significant, β = 0.044, *p* = 0.234, 95%CI = [−0.025, 0.112]. The indirect effect was statistically significant, β = 0.017, *p* < 0.005, 95%CI = [0.005, 0.037]. T1 Dire to T3 MIL-p was not significant (β = 0.044, *p* = 0.210).

For Emotional, the direct effect was not significant, β = 0.006, *p* = 0.218, 95%CI = [−0.066, 0.080]. The indirect effect was statistically significant, β = 0.018, *p* < 0.005, 95%CI = [0.006, 0.040]. T1 Emotional to T3 MIL-p was not significant (β = 0.006, *p* = 0.861). T2 Emotional to T3 PSS was not significant (β = 0.070, *p* = 0.054). T2 PSS to T3 Emotional was not significant (β = 0.072, *p* = 0.058). These results support our hypothesis that perceived social support mediates the relationship between meaning in life and Anonymous, Compliant, Altruistic, Dire, and Emotional prosocial behavior, but not Public prosocial behavior.

The results also support our hypothesis that the relationship between perceived social support and meaning in life is bidirectional. In all six models, the path from T2 PSS to T3 MIL-P, T1 MIL-P to T2 PSS, and T2 MIL-P to T3 PSS were all statistically significant.

## 4. Discussion

We built on previous work to better understand how prosocial behaviors are associated with meaning in life. Using three waves of longitudinal data of Chinese students, we hoped to shed light on the mediating role of perceived social support in the meaning-providing function of multidimensional prosocial behavior. The result showed that during the COVID-19 pandemic, there was a slight decrease in both perceived social support and MIL-p and an increase in prosocial behavior, with the exception of Public. Perceived social support mediated the relationship between meaning in life and five dimensions of prosocial behavior, i.e., Altruistic, Anonymous, Compliant, Emotional, and Dire, but not Public. The direct effect of each dimension of prosocial behavior on meaning in life was not significant. We also found bidirectional relationships between support and meaningfulness.

### 4.1. The mediation role of perceived social support

All direct effects were non-significant. All six dimensions of prosocial behavior at T1 did not predict meaning in life at T3 (Altruistic had marginal significance). All five other dimensions of prosocial behavior (except Public) increased perceived social support and consequently contributed to meaning in life. This is generally consistent with prior research (16, 17). Collectivism promotes “we” consciousness, collective identity, emotional dependency, group unity, and duty and responsibility (80). Prosocial behavior toward other individuals strengthens social identity and group attitudes. Such attachments and support may help increase a person's sense of self-worth and lower anxiety regarding personal coping efficacy (81) Individuals may also be able to create a more profound sense of purpose or meaning through their social relationships (82). Thus, social connections, as captured by social support in this study, may be the underlying theoretical link between prosocial behavior (namely Altruistic, Anonymous, Compliant, Emotional, and Dire), and meaning in life. From the standpoint of positive psychology, social support not only acts as a buffer against adversity, it also contributes to a flourishing and meaningful life in the absence of adversity. This support may be acquired through prosocial action, which is under one's own control and is self-determined. Individuals can turn to other-oriented good acts, integrate themselves into the community, be kind to others, and ultimately achieve high-quality relationships and a meaningful life.

In contrast to the other prosocial behaviors, Public did not significantly predict perceived social support. Help undertaken in front of others is connected with self-oriented motives (e.g., to obtain the approval and respect of others). Existing studies have revealed that the socialization of other-oriented prosocial behavior differs significantly from that of selfishly driven prosocial behavior and prosocial activity motivated by the desire for approval (40, 83, 84). Although researchers have emphasized that social desirability concerns are not necessarily incompatible with prosocial behavior (22). Our findings demonstrate that self-directed prosocial behavior may not result in high-quality interpersonal connections.

### 4.2. Bidirectional relationship of perceived social support and meaning in life

The reciprocal relationships between meaning and interpersonal relationships shown in our results have been found in prior research (58). According to Baumeister's Need to Belong Theory (1995), as well as a motivational hierarchy (85), a sense of belonging is a basic human need that motivates the formation and maintenance of interpersonal relationships, and it lays the foundations for forming meaning in life. The belief that life is meaningful also contributes to the formation of interpersonal bonds and high-quality interpersonal relationships (55). Such studies and ideas show the critical role of social relationships in developing meaning in life. This implies that a prerequisite for developing meaning in life is to establish a good interpersonal relationship. Therefore, the fact that a higher sense of meaning in life can predict greater perceived social support is no new revelation.

### 4.3. The time differences of studied variables during COVID-19

The relationship between suffering and prosocial behavior has been inconsistent and controversial depending on the context (86). The present study found that Altruistic and Dire prosocial behavior were significantly elevated during the COVID-19 outbreak. This means individuals would be more likely to help others during a severe virus outbreak. Shared painful or traumatic experiences can facilitate a feeling of closeness and connection (87), especially for pure altruism (86). Therefore, hardship promotes cooperation as a strategy to overcome shared adversities (88, 89). Other dimensions of prosocial behavior, like Compliant, Emotional, and Anonymous exhibited the same upward tendency; it was not statistically significant but was, nonetheless, worthy of note.

The consistent decline in perceived social support and meaning in life suggests that the effects of COVID-19 on mental health are likely to be profound and long-lasting. Social distancing and self-isolation reduced social relations and increased the crisis of loneliness, loss, anxiety (90, 91), sense of meaning less, and suicidal ideation (92). The COVID-19 outbreak stopped us from exploring the world, pursuing our goals, and interacting with loved ones. It shifted our perspective on life and forced people to consider the purpose of their lives, putting them in an incomprehensible and unpredictable situation that led to meaninglessness. Has the COVID-19 pandemic changed us permanently? The long-term impacts and dynamics of coronavirus's impact on people's mental health must be explored and monitored in more depth.

## 5. Limitations

Despite the advantages of our three-wave design, our study has some limitations that pave the way for future research. First, at T1, we did not collect perceived social support. Future research can improve this design and include all measures at all time points. Second, we relied on self-reported data. Given that people naturally believe themselves to be prosocial (93), and that people can overestimate their actual prosocial action due to self-image concerns, it is likely that self-reports are consistently inflated. Future research can theorize and test a more nuanced view in different cultural contexts, focusing on specific dimensions of prosocial behaviors.

## 6. Conclusion

We discovered that during difficult times, such as the COVID-19 pandemic, people act more pro-socially, which can increase perceived social support, build a good social network, and ultimately increase life meaning. During a crisis, such as the COVID-19 pandemic, these practices may alleviate loneliness while also increasing social support and meaning in life. To regain psychological equilibrium and a sense of meaning, we can engage in prosocial activities.

## Data availability statement

The raw data supporting the conclusions of this article will be made available by the authors, without undue reservation.

## Ethics statement

The studies involving human participants were reviewed and approved by the Institutional Review Board at the University of Guangxi Normal University. The patients/participants provided their written informed consent to participate in this study.

## Author contributions

YH: investigation, formal analysis, writing – original draft, and writing – review and editing. QL: investigation and data curation. OT: writing – review and editing. QH: review and editing and supervision. SZ: conceptualization, methodology, review and editing, supervision, project administration, and funding acquisition. All authors contributed to the article and approved the submitted version.
